# YAP as a therapeutic target in esophageal squamous cell carcinoma: insights and strategies

**DOI:** 10.1080/07853890.2025.2536200

**Published:** 2025-07-22

**Authors:** Weilong Wang, Tingmin Chang, Lingchao Wang, Rafeezul Bin Mohamed, Nor Hazwani Binti Ahmad, Xiumin Li

**Affiliations:** a Department of Gastroenterology, the First Affiliated Hospital of Xinxiang Medical University, Xinxiang, Henan Province, PR China; b Department of Biomedical Science, Advanced Medical and Dental Institute, Universiti Sains Malaysia, Kepala Batas, Pulau Pinang, Malaysia; c Xinxiang Key Laboratory for Molecular Therapy of Cancer, Xinxiang Medical University, Xinxiang, Henan Province, PR China; d Department of Pediatric, The First Affiliated Hospital of Xinxiang Medical University, Xinxiang, Henan Province, PR China

**Keywords:** Esophageal squamous cell carcinoma, hippo pathway, YAP, therapeutic strategy

## Abstract

**Background:**

Yes-associated protein (YAP) is a core component of the Hippo pathway, which functions as an oncogene in various cancers. However, emerging evidence has shown that YAP can also act as a tumor suppressor. Therefore, understanding the function and molecular mechanism of YAP is crucial for developing YAP-targeted drugs in tumors.

**Methods:**

A comprehensive literature review was conducted. The review mainly includes the post-translational modification, the regulatory mechanisms and function of YAP in esophageal squamous cell carcinoma (ESCC).

**Results:**

YAP undergoes various post-translational modifications (PTMs), including phosphorylation, ubiquitination, acetylation and others, which critically regulate its protein stability and transcriptional activity in multiple tumors, particularly ESCC. YAP is highly expressed in ESCC tissues, with its aberrant activation closely correlated with poor prognosis in patients. Additionally, YAP is involved in the progression of ESCC, including tumor migration, invasion, proliferation, cell stemness, apoptosis, therapeutic resistance, and immunity. In ESCC, YAP has been confirmed to be regulated by multiple upstream regulators, such as E3 ubiquitin ligases and kinases, thereby influencing the ESCC progression. However, there are still few drugs available clinically for YAP-targeted therapy, which requires further research. In this review, we systematically synthesize the biological roles and regulatory mechanisms of YAP in ESCC and outline potential research directions for YAP-targeted therapies, aiming to provide novel insights for precision medicine in ESCC.

**Conclusion:**

YAP is closely correlated to ESCC progression, and it could be a promising target for ESCC treatment.

## 1. Background

According to GLOBOCAN 2020, esophageal cancer ranks seventh in incidence and sixth in mortality rates among cancers worldwide [1]. China has the highest number of new cases of esophageal cancer in the world, followed by India, Japan, and Bangladesh [2]. Esophageal cancer mainly has two subtypes including esophageal squamous cell carcinoma (ESCC) and esophageal adenocarcinoma (EAC) [3]. Globally, ESCC accounts for 88% of all esophageal cancer cases and contributes significantly to the overall disease burden [4]. Several factors are correlated with ESCC, such as genetic inheritance, HPV infection, riboflavin deficiency, smoking, consumption of hot beverages, alcohol use, and achalasia [5,6]. Due to the aggressive characteristics and challenges of early detection, over 75% of patients with ESCC are typically diagnosed at an advanced stage, resulting in a 5-year survival rate of less than 20% [7,8]. Furthermore, although new methods have been proposed for the treatment of esophageal cancer in recent years, the newly developed treatments for esophageal cancer have little clinical impact and have only resulted in modest gains in survival rate. Despite these circumstances, scientists are still optimistic about esophageal cancer therapies. Some types of targeted therapy for esophageal cancer treatment have been approved by the United States Food and Drug Administration (FDA) in the past decade, and there are still several drugs pending approval [9]. As a result, it is necessary to explore new targets for ESCC treatment.

YAP (YES-associated protein) is the key downstream effector protein of the Hippo pathway, playing a significant role in regulating organ size, cell proliferation, differentiation, and cancer progression [10,11]. When the Hippo pathway is inhibited or dysregulated, YAP is activated and translocated into the nucleus where it acts as a transcriptional co-activator. In this nuclear location, YAP interacts with transcription factors such as TEADs (TEA domain family members) to promote the expression of genes involved in cell proliferation, migration, and survival in gastric cancer, hepatocellular carcinoma, ESCC, etc [12–17].

Paradoxically, YAP can also exert tumor-suppressive effects under certain conditions. For instance, in some contexts, YAP has been shown to induce the expression of genes involved in apoptosis, thereby inhibiting tumor growth. For example, Levy et al. discovered that YAP can compete with Itch (a ubiquitin E3 ligase) for binding to p73, thereby preventing Itch-mediated degradation of p73. This interaction induces apoptosis in cisplatin-treated HCT116 cells [18]. Similarly, YAP has been found to cause apoptosis in response to DNA damage by increasing p73 transcription factor function in apoptotic gene promoters [19,20].

The dual functions of YAP in cancer indicate the complexity of its regulatory mechanisms and the context-dependent nature of its activity. Understanding the specific molecular context in which YAP functions is crucial for developing targeted therapeutic strategies for cancer treatment.

## 2. Molecular structures and function of hippo/YAP

### 2.1. Molecular structures of YAP

**Figure 1.**
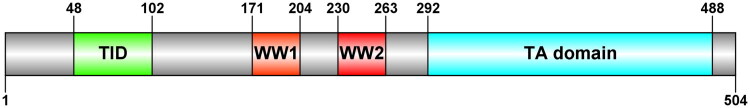
YAP protein domain structure. The TID domain is responsible for binding transcription factors; the WW domain serves as protein-protein interaction; the TA domain mediates transcription activation.

YAP was first discovered in chickens as a binding protein for the non-receptor tyrosine kinase YES1 in 1994 [21]. YAP has three important conserved domains including the TID domain (TEAD interaction domain) at the N-terminus, the WW domain, and the TA domain (transcription activation domain) in the C-tail. The WW domain commonly determines the localization and activity of the YAP protein by recognizing the PY motif (PPxY, P for proline, Y for tyrosine, x for any amino acid). In addition, its TID domain and transcriptional activation domain can bind a series of transcription factors and enhance their activity (Figure 1). Notably, YAP cannot bind DNA directly as it is a transcription co-activator, whereas it interacts with other transcription factors to regulate target gene expression. The primary transcription factors that bind to YAP are TEAD family members including TEAD1, TEAD2, TEAD3, and TEAD4. For example, YAP enhanced the transcriptional expression of PKMYT1 by binding directly to the PKMYT1 promoter through TEAD1-mediated mechanisms [22]. This association is linked to a poor prognosis for bladder cancer. A previous study also reported that YAP/TEAD1 can regulate NAIP at the transcriptional level [23]. Furthermore, many studies have shown YAP interacts with TEADs to activate expression of the target genes and regulate cancer progress [24–28]. Besides, other transcription factors (AP-1, RUNXs) have also been uncovered to interact with YAP in cancer [29–32].

### 2.2. Functions of the hippo/YAP pathway

**Figure 2.**
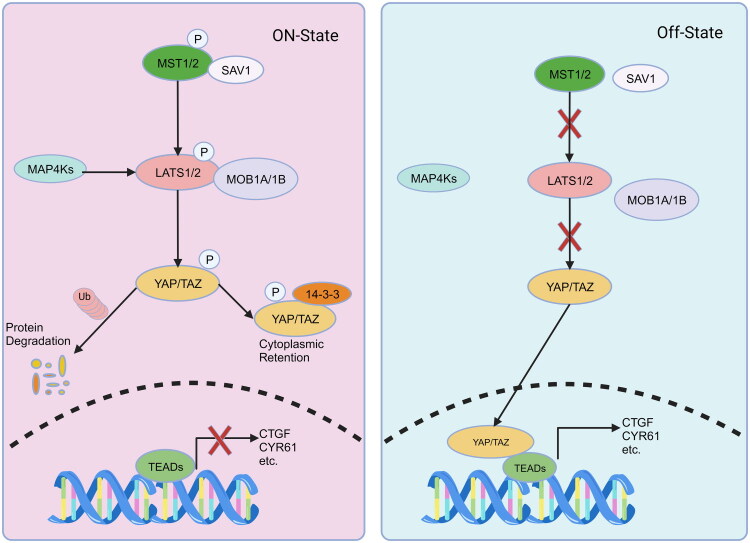
Core components of the hippo pathway in mammalian cells. When hippo signaling pathway is activated, MST1/2 and SAV1 phosphorylate LATS1/2 and MOB1, thereby promoting YAP/TAZ phosphorylation. Activated YAP/TAZ binds to 14–3–3 and is degraded *via* the ubiquitin-proteasome pathway in the cytoplasm. When hippo signaling pathway is inactivated, YAP/TAZ translocate to the nucleus, forming a complex with TEADs to activate transcription of target genes.

The Hippo signaling pathway is an extremely evolutionarily conserved pathway in different species. It was first discovered in *Drosophila melanogaster* as a tumor suppressor pathway [33]. Recently, a systematic profiling of 9,125 tumor samples revealed a widespread dysregulation of Hippo pathway components in multiple human cancer types, including glioma, colorectal cancer, and endometrial cancer, which contributes to cancer development and tumorigenesis [34]. The Hippo signaling pathway can be activated by several factors including cell density, G-protein-coupled receptors (GPCRs), cell junctions, mechanical stress, cellular stress, obesity [35]. The core components of the Hippo pathway in mammals includes a kinase cascade, LATS1/2(large tumor suppressor homolog1/2) and their adapter proteins MOB1 (mps 1 binder kinase activator-1), MST kinases1/2(the mammalian STE20-like protein 1/2) with their adapter protein SAV1 (Salvador homolog-1) [36]. When the Hippo signaling pathway is activated, MST1/2 are phosphorylated, and then phosphorylate LATS1/2 which also can be phosphorylated by MAP4Ks [37]. LATS1/2 enzymes become active and phosphorylate YAP/TAZ. This leads to the retention of YAP/TAZ in the cytoplasm through a process involving 14–3–3 proteins [38]. Additionally, YAP/TAZ undergo degradation in the proteasome through a ubiquitination-dependent. When the Hippo pathway is inactive, YAP/TAZ undergo dephosphorylation and enter the nucleus [39]. Once there, they will bind to TEADs, which leads to the activation of downstream target genes (such as CTGF, CYR61, etc) *via* transcription (Figure 2).

## 3. Posttranslational modifications of YAP in cancer

**Figure 3.**
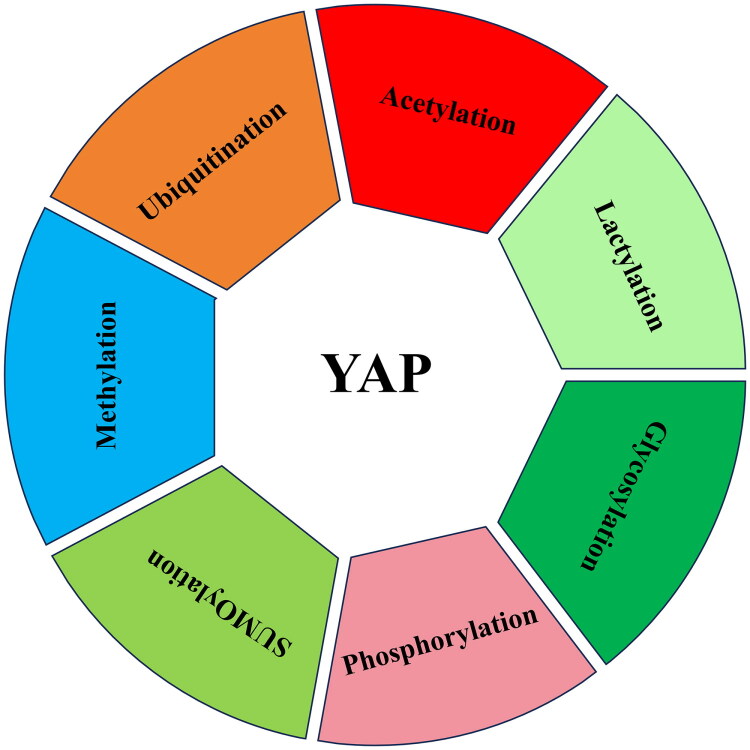
Post-translational modifications of YAP. YAP can be regulated by several post-translational modifications including ubiquitination, methylation, acetylation, glycosylation, phosphorylation, SUMOylation, and lactylation.

YAP is regulated by several posttranslational modifications, such as phosphorylation, methylation, glycosylation, acetylation, SUMOylation, ubiquitination, and lactylation (Figure 3).

YAP undergoes phosphorylation more frequently than any other types of posttranslational modification. The phosphorylation of YAP can be divided into Hippo-dependent ways and Hippo-independent ways. For the Hippo-dependent ways, activated LATS1/2 can phosphorylate YAP on five Serine(S) residues (S61, S109, S127, S164, and S381, respectively) and inactivate YAP [40]. For the Hippo-independent way, Brent et al. have documented the capacity of mTORC2 to directly phosphorylate YAP at S436, resulting in a beneficial regulation of YAP activity [41]. Furthermore, the important residues on YAP are S127 and S381 which are closely related to inhibiting the Hippo signaling. Phosphorylation of S127 favors binding to the 14-3-3 protein and cytoplasmic retention, whereas phosphorylation of S381 is predisposed to CK1-mediated phosphorylation and subsequent degradation *via* the proteasome system. However, little is known the phosphorylation of YAP at other sites. Maybe multiple sites can work together to maintain the normal function of YAP. Therefore, more investigations are required.

Another common posttranslational modification of YAP is ubiquitination and deubiquitination *via* the proteasome-dependent pathway. Ubiquitination regulates a variety of physiological activities and is inhibited by deubiquitination. There is a strong link between dysregulation *of* ubiquitination and deubiquitination and the onset and progression of tumors [42]. Yan et al. reported that OUTB1 could interact with the YAP protein and remove ubiquitin molecules from YAP at multiple lysine(K) sites (K90, K280, K342, K494, and K497) [43]. This interaction ultimately prevents the degradation of YAP in gastric cancer. Additionally, YAP could also be degraded by ZNF213 *via* K48-linked polyubiquitination at lysine sites (K252, K254, K321, and K497) on YAP in breast cancer, thereby inhibiting YAP target gene expression [44]. Different from protein degradation, another study discovered that tumor necrosis factor receptor-associated factor 6 (TRAF6) initiates YAP K63 polyubiquitination at the lysine 252 site (K252), thus disrupting the interaction between YAP and angiomotin and resulting in enhanced YAP nuclear translocation [45].

YAP can also be modified by methylation, deacetylation, O-GlcNAcylation and lactylation. For instance, Fang et al. described that SET1A methylates YAP at the lysine 342 (K342) site, and causes nuclear retention of YAP and increased YAP activity [46]. In terms of deacetylation, PXR can promote liver enlargement and regeneration in mice through Sirt2-driven YAP deacetylation and K63-linked YAP polyubiquitination [47]. A recent investigation reported that Sirt1 facilitated the deacetylation of YAP, leading to the export of YAP from the nucleus and its subsequent destruction [48]. On the other hand, O-GlcNAcylation has also been reported by several studies in recent years. Peng et al. showed that YAP undergoes O-GlcNAcylation by OGT at S109, subsequently interrupting its interaction with LATS1, therefore activating YAP transcriptional activity and promoting tumorigenesis [49]. Another report revealed that OGT (O‑GlcNAc transferase) knockdown decreases the O‑GlcNAcylation of YAP by mass spectrometric analysis, leading to YAP phosphorylation and inhibits YAP nucleus import in endometrial cancer [50]. SUMOylation is achieved by covalently attaching the small ubiquitin-like modifier (SUMO) to a protein, which plays an important role in nuclear cytoplasmic transport, transcriptional regulation, protein stability, stress response, and cell cycle progression [51]. In recent years, the SUMOylation modification of YAP has also been confirmed by different studies. For instance, CBX4 can promote YAP protein SUMOylation, thus inhibiting cell aging and desensitizing cells to chemotherapy in gastric cancer [52]. Lactylation is a newly discovered metabolism-associated post-translational modification, which refers to the enzymatic transfer of a lactyl group derived from lactic acid to the lysine residues of proteins. Lactylation mainly is involved in regulating gene transcriptional activity, protein activity, and interactions. Recent studies have shown that the YAP protein is also subject to lactylation modification. For instance, a recent study showed that K90 lactylation of YAP regulates the nuclear retention of YAP by disrupting its interaction with the export receptor CRM1, thereby promoting the malignancy of hepatocellular carcinoma [53]. Importantly, most mammalian proteins are modified by multiple post-translational modifications (PTMs), which can crosstalk with each other, a process known as PTM crosstalk. The PTM crosstalk can integrate multiple signals. Given that the amino group of lysine can undergo various modifications, including, methylation, ubiquitination, lactylation, and ubiquitin-like modifications, this may lead to competitive PTM crosstalk, in which different PTMs compete to modify the same lysine residue. For example, the YAP K342 site can be modified by OTUB1 and SET7, while the K494 site can be regulated by OTUB1 and SET1A. Table 1 summarize the PTMs at different sites of YAP. Taken together, YAP is regulated by different PTMs in a context-dependent manner. Its function varies depending on the modification site and type. It may be the combination of various modifications that can maintain homeostasis in the organisms. Furthermore, it is necessary to explore the PTM sites before developing new drugs for targeting YAP in the future.

**Table 1.** Posttranslational modification sites of YAP.

Amino acid residues	Post-translational modification types	Enzyme/kinase	Reference
Serine 61	Phosphorylation	LATS1/2; AMPK	LATS1/2 [40]; AMPK [165]
Lysine 90	DeUbiquitination; Ubiquitination	OTUB1; PARK2	OTUB1 [43]; PARK2 [102]
Serine 94	Phosphorylation	AMPK	AMPK [54]
Lysine 97	SUMOylation	CBX4	CBX4 [52]
Serine109	Phosphorylation; O-GlcNAcylation	LATS1/2; OGT	LATS1/2 [40]; OGT [49]
Threonine 119	Phosphorylation	JNK1/2; CDK1	JNK1/2 [166] CDK1 [148]
Serine 127	Phosphorylation	LATS1/2	LATS1/2 [40]
Serine 164	Phosphorylation	LATS1/2	LATS1/2 [40]
Tyrosine 188	Phosphorylation	unknown	27428284 [167]
Threonine 241	O-GlcNAcylation	OGT	OGT [168, 169]
Lysine 252	Ubiquitination	ZNF213; TRAF6	ZNF213 [44] TRAF6 [45]
Lysine 254	Ubiquitination	ZNF213	ZNF213 [44]
Serine 274	Phosphorylation	ERK	ERK [170]
Lysine 280	SUMOylation; DeUbiquitination	CBX4; OTUB1	CBX4 [52]; OTUB1 [43]
Serine 289	Phosphorylation	CDK1	CDK1 [148]
Lysine 321	Ubiquitination	ZNF213	ZNF213 [44]
Lysine 342	DeUbiquitination; Methylation; Demethylation	OTUB1; SET1A; LSD1	OTUB1 [43] SET1A [46] LSD1 [46]
Serine 352	Phosphorylation	ERK	ERK [170]
Tyrosine 357	Phosphorylation	Src	32246402 [171]
Serine 367	Phosphorylation	CDK1	24101154 [148]
Serine 381	Phosphorylation	LATS1/2	LATS1/2 [40]
Serine 397	Phosphorylation; dephosphorylation	CDK7; PP1A	CDK7 [82] PP1A [172]
Serine 403	Phosphorylation	IKKε	IKKε [173]
Serine 436	Phosphorylation	mTORC2	mTORC2 [41]
Lysine 494	Methylation; DeUbiquitination; Acetylation; Deacetylation	OTUB1; Set7; CBP/P300; SIRT1	OTUB1 [43]; Set7 [174]; CBP/P300 [175]; SIRT1 [175]
Lysine 497	DeUbiquitination; Ubiquitination; Acetylation; Deacetylation	OTUB1; ZNF213; CBP/P300; SIRT1	OTUB1 [43] ZNF213 [44] CBP/P300 [175]; SIRT1[175]

## 4. YAP in esophageal squamous cell carcinoma

**Figure 4.**
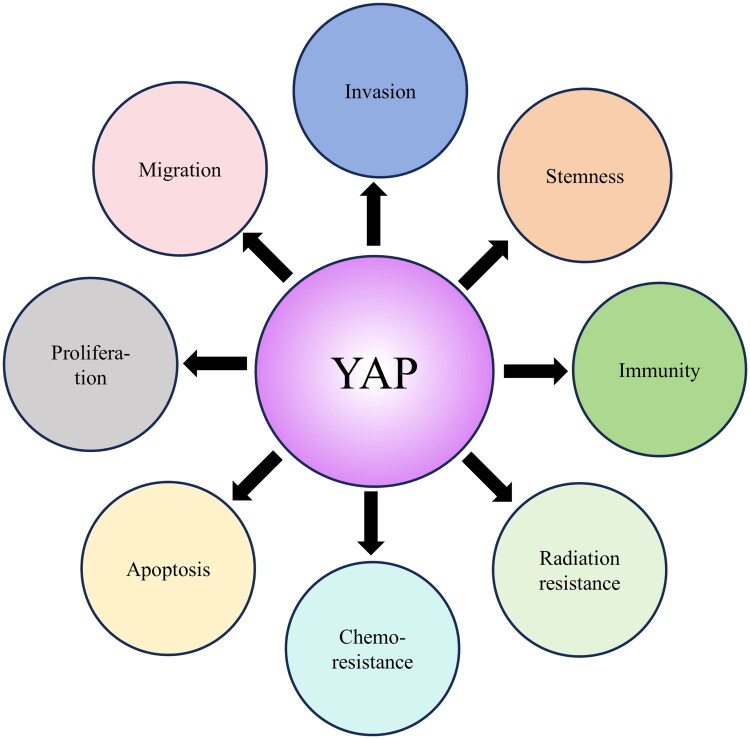
The functions of YAP in ESCC. YAP exerts various functions in ESCC, such as migration, invasion, stemness, immune evasion, radiation resistance, chemoresistance, apoptosis, and proliferation.

Although YAP activation is frequently observed in tumors, the mutation frequency of YAP is relatively low, including in ESCC [34]. YAP has been shown to be closely associated with the progression of ESCC. For instance, Gao et al. reported that mutations in FAT1, FAT2, FAT3, FAT4, and AJUBA dysregulate the Hippo pathway, consequently leading to hyperactivation of YAP in ESCC [55]. Previous studies have shown that YAP could be an independent prognostic marker in ESCC, with increased YAP expression associated with a worse prognosis [56–58]. Furthermore, several studies have demonstrated that YAP is overexpressed in ESCC and could control cancer cell proliferation, migration, invasion, apoptosis, stemness, and sphere formation *via* various downstream factors [56,59–67] (Figure 4).

### 4.1. Function of YAP in ESCC migration, invasion, proliferation, and apoptosis

Metastasis and invasion are significant hallmarks of cancer which are closely related to the cancer progression [68]. Epithelial-mesenchymal transition (EMT) is a biological process that occurs when cells lose their epithelial traits and gain mesenchymal characteristics. The process of EMT is associated with various cancer-related phenomena, such as the genesis of cancer, the maintenance of cancer stem cells, the migration and invasion of cells, and resistance to therapies [69–72]. It is reported that YAP contributes to EMT progression in ESCC. For instance, YAP knockdown could significantly inhibit migration and invasion which are related to EMT markers N-cadherin, vimentin, and E-cadherin in Eca109 and Kyse150 cell lines [58]. Furthermore, the proliferation, invasiveness, and ability to form spheres are notably diminished following the silencing of YAP using two siRNAs in EC9706 and Eca109 cell lines. The underlying mechanism is that YAP binds TEAD, thereby leading to an elevation in IRS2 expression [61]. Furthermore, YAP can regulate cell proliferation and apoptosis through different mechanisms. A study showed YAP depletion negatively influences cell proliferation of the KYSE170 cell line mainly in the G0–G1 phase through CDKN1A/p21 and BIRC5/survivin [56]. However, it is still uncertain whether these effects are direct or indirect consequences of YAP. Moreover, by TEAD4‐mediated direct binding to KIF4A promoter, YAP triggers KIF4A transcriptional expression, thereby fostering the ESCC proliferation [59].

Interestingly, Cui et al. proposed that inhibiting the YAP gene leads to a substantial decrease in ECA-109 cell proliferation and triggers cell death. However, this inhibition does not have any significant impact on the cell cycle [65]. Consequently, far more research is needed on whether YAP regulates the ESCC cell cycle or not.

### 4.2. Function of YAP in ESCC cell stemness

YAP regulates several downstream factors and exhibits oncogenic functions in different aspects of ESCC. Previous studies have shown that cancer stem cells are considered to initiate and sustain tumor growth. Song et al. identified YAP-driven SOX9 expression as a critical event in the acquisition of cancer stem cell (CSC) properties [73]. Specifically, YAP upregulates direct expression of SOX9 *via* transcription at the SOX9 promoter and endows CSC properties onto a wide variety of non-transformed cell types of gastrointestinal origin, including primary esophageal epithelium cells, immortalized embryonic liver cells as well as ESCC cells. Another similar study also provided evidence that genetic hyperactivation of YAP disrupts the YAP-SOX9 feedback loop and gives rise to characteristics resembling cancer stem cells in ESCC [60].

### 4.3. Function of YAP in ESCC treatment

Moreover, YAP is also closely related to the treatment of ESCC. For example, in esophageal cancer cells, YAP promoted CDK6 transcription and expression, leading to radiation resistance, whereas CDK6 knockdown abrogated YAP-mediated radiation resistance. Moreover, compared to mice treated with CA3(YAP inhibitor) or LEE001(CDK6 inhibitor) alone, mice treated with LEE001 and CA3 in combination have a more significant reduction in tumor volume. The combination of CA3 and LEE001 has stronger anticancer activity in radiation-resistant esophageal cancer mice [63]. In addition, Song et al. discovered that YAP can increase Epidermal growth factor receptor (EGFR) expression, resulting in resistance to 5-FU and docetaxel, whereas YAP knockdown renders ESCC cells susceptible to these [64]. Additionally, our group’s latest research confirmed that verteporfin treatment can reduce the tumor formation in the 4-NQO-induced esophageal cancer mouse model. On the other hand, YAP has the potential to enhance the production of CD24 *via* transcriptional regulation, a newly discovered mechanism that regulates the innate immune response in cancer [12]. Moreover, suppressing YAP activity has been shown to diminish the accumulation of myeloid-derived suppressor cells (MDSCs) within the tumor microenvironment, thereby contributing to improved radiosensitivity in ESCC [74]. In conclusion, focusing on YAP could be a potentially effective approach to improve the prognosis of patients with ESCC.

These results indicate that YAP serves as an oncogene in ESCC. However, a recent study from the Kuo group reported that YAP acts as a tumor suppressor in ESCC. Experiments conducted *in vitro* and *in vivo* demonstrated that knockdown of YAP by shRNA could increase proliferation, and invasion ability in two ESCC cell lines (CE81T(1-0) and KYSE-170, respectively). YAP may function as suppressor by transcriptionally regulating pro-tumor TAZ expression in esophageal cancer [75]. The possible reason for the dual roles of YAP is that the genetic background of cell lines is differ. Due to the limited research on the anticancer function of YAP in ESCC, more studies are needed to verify whether YAP exerts tumor-suppressive functions. Overall, YAP exerts different functions by regulating different downstream factors in ESCC (Table 2).

**Table 2.** The downstream of YAP in ESCC.

Gene symbol	Model	Mechanism Summary
N-cadherin, vimentin, and E-cadherin	Eca109 and Kyse150 cell lines.	Knockdown of YAP can reduce expression levels of vimentin and N-cadherin, whereas that of E-cadherin was upregulated, thus inhibiting ESCC progression [57].
IRS2	EC9706 and Eca109 cell lines.	YAP-TEAD activates the JNK/c-Jun pathway to upregulate IRS2, thus promoting ESCC progression [60].
p21 and survivin	Patient’s tumor tissue; KYSE170 and KYSE1240 cell lines.	Depletion of YAP hinders cell proliferation mostly in the G0–G1 phase and resulted in transcription upregulation of CDKN1A/p21 but a transcription downregulation of BIRC5/survivin [55].
SOX9	Patient’s tumor tissue; nude mice; KATO-TN, EC9706, and TE-1 cell lines.	YAP transcriptionally activates SOX9 *via* TEAD1 mediated binding, then driving stemness and malignant progression of ESCC [59].
CDK6	Nude mice; KATO-TN and YES-6 cell lines.	YAP-mediated CDK6 activation confers radiation resistance in ESCC [62].
KIF4A	Eca109 and TE-7 cell lines.	YAP activates KIF4A transcriptional expression by TEAD4‐mediated direct binding to the KIF4A promoter, thereby promoting the proliferation of ESCC [58].
EGFR	Nude mice; KATO-TN and YES-6 cell lines.	YAP positively regulates sustained EGFR expression at the transcription level through a TEAD binding site, then inducing resistance to 5-FU and docetaxel in ESCC [63].
TAZ	CE81T(1-0) and KYSE-170 cell lines.	YAP acts as a suppressor and inhibits the expression of TAZ in ESCC through transcriptional and translational regulation [74].
CD24	Patient’s tumor tissue; nude mice; EC9706 and ECA109 cell lines.	YAP promotes CD24 expression *via* transcription, thereby driving immune evasion *via* macrophage in ESCC phagocytosis [12].
HIF-1α	KYSE150 cell line	YAP over-expression could increase HIF-1α protein level, thus increasing enhancing radiotherapy sensitivity in ESCC [73].

## 5. Regulation of YAP in ESCC

**Figure 5.**
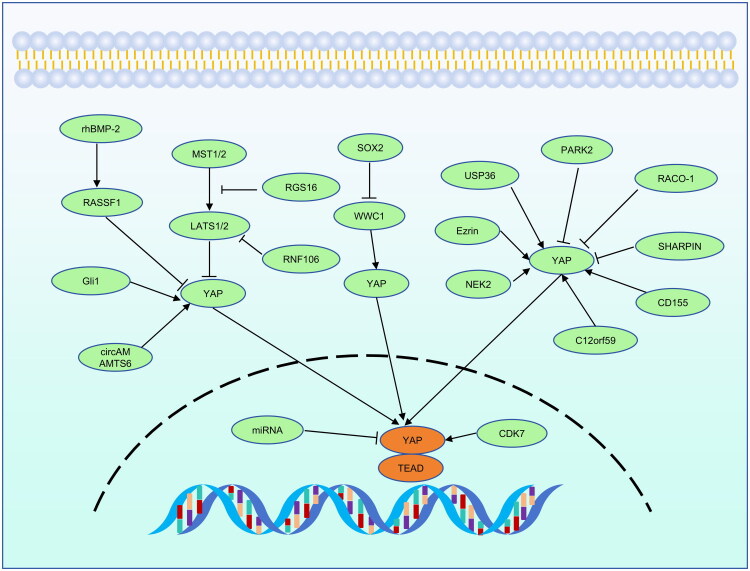
Upstream regulatory factors of YAP in ESCC. In ESCC, YAP activity can be inhibited by several factors including RhBMP-2, miRNAs, SOX2, PARK2, RACO-1, SHARPIN. Moreover, YAP can be activated by Gli1, circAMAMTS6, CDK7, RGS16, RNF106, USP36, ezrin, NEK2, CD155 and C12orf59.

YAP can be modulated by several factors and posttranscriptional modifications. Furthermore, studies have demonstrated that the Hippo pathway also could crosstalk with other signaling pathways including transforming growth factor-β (TGF-β) pathway, AMPK pathway, MAPK signaling, WNT-β-catenin pathway to modulate YAP expression and activity (Figure S1) [37,54,76,77]. This section will focus on the regulation of YAP in ESCC (Figure 5).

In human ESCC, activation of YAP could be directly or indirectly modulated by different intracellular factors. For example, rhBMP-2 (recombinant human bone morphogenetic protein-2), commonly used as a spine graft substitute [78,79], was reported to increase p-YAP protein levels, which could be reversed by RASSF1 knockdown, consequently inhibiting proliferation in two different ESCC cell lines [80]. Zhang et al. found that RGS16 promotes YAP activity by disrupting the interaction between LATS1 and MST1 in KYSE410 and KYSE150 cell lines [81]. In addition, lv et al. discovered that cyclin-dependent kinase 7 (CDK7) activates nuclear YAP at S127 and S397 sites, thereby increasing its transcriptional activity [82]. This, in turn, stimulates the production of D-lactate dehydrogenase (LDHD) protein and promotes cancer stem cell-like features in ESCC [82]. Moreover, overexpression of CD155 decreased the level of phosphorylated YAP and promoted radiotherapy resistance and malignancy of ESCC in the Eca109 cell line [83]. Furthermore, Chai et al. demonstrated that SOX2 can increase the migration and invasion ability of ESCC by indirectly driving YAP activation *via* WWC1 in Eca109 and KYSE150 cell lines [84]. Another study revealed that C12orf59 promotes YAP activation. However, its underlying mechanism is still unclear. It possibly stimulates YAP activity *via* the upstream signaling of the Hippo pathway [85]. Additionally, a preclinical study has shown that the total protein level of YAP increased after slicing Ezrin, indicating that further research is needed to understand the regulatory mechanism involved [86]. Besides, Gli1, a key transcription factor of the Hedgehog signaling pathway, has been correlated with esophageal cancer progression. In ESCC, Gli1 and YAP were overexpressed and were associated with poor prognosis [87]. Mechanistically, Gli1 interactd with YAP and decreased the phosphorylation of YAP at the S127 site in LATS1-independent manner [88]. Furthermore, recent research from Su’s group demonstrated that NEK2 enhances ESCC migration and proliferation ability by stabilizing YAP through phosphorylation at the Threonine 143 site [89].

A number of noncoding RNAs (ncRNAs) have also been reported to be involved in the regulation of YAP. For example, miR-624 might target ARRDC3 and limit its expression, weakening YAP degradation mediated by ARRDC3-YAP coprecipitation and increasing YAP expression in ESCC cells [90]. Moreover, miR-141 targeted at the 3′-untranslated region of YAP, resulting in the suppression of YAP expression [91]. Similar to miR-141, it was reported that miR-920 can also directly target and regulate YAP gene expression in two independent studies [92,93]. Furthermore, circADAMTS6 was confirmed to promote the progression of ESCC by regulating AGR2 and YAP. Mechanistically, on the one hand, circADAMTS6 knockdown significantly increases the YAP protein level. On the other hand, it decreases the level of phosphorylated YAP simultaneously [94].

Ubiquitination is also a vital mechanism that regulates YAP expression and activity [95–97]. In particular, RNF106 improved YAP activity *via* facilitating LATS2 K48-linked ubiquitination and degradation, thus promoting esophageal squamous cell carcinoma progression [98]. In addition, USP36 was associated with the YAP protein and enhanced YAP protein stability by blocking the K48-linked polyubiquitination of YAP, thus facilitating proliferation, migration, and invasion of ESCC [99]. In recent years, our group has identified several new E3 ubiquitin ligases targeting YAP in ESCC cells, including RACO-1, SHARPIN, and PARK2 [100–102]. Specifically, RACO-1 and SHARPIN inhibit migration and invasion by promoting YAP protein degradation *via* K48-linked polyubiquitination. PARK2 can interact with YAP through its RING domain, thus promoting YAP degradation in a ubiquitination-dependent manner. Of note, our group identified that PARK2 facilitates YAP protein ubiquitination and degradation at the K90 site which may be a potential target for the treatment of ESCC in the future [102].

## 6. Preclinical studies demonstrating the efficacy of YAP-targeting drugs in ESCC models

**Figure 6.**
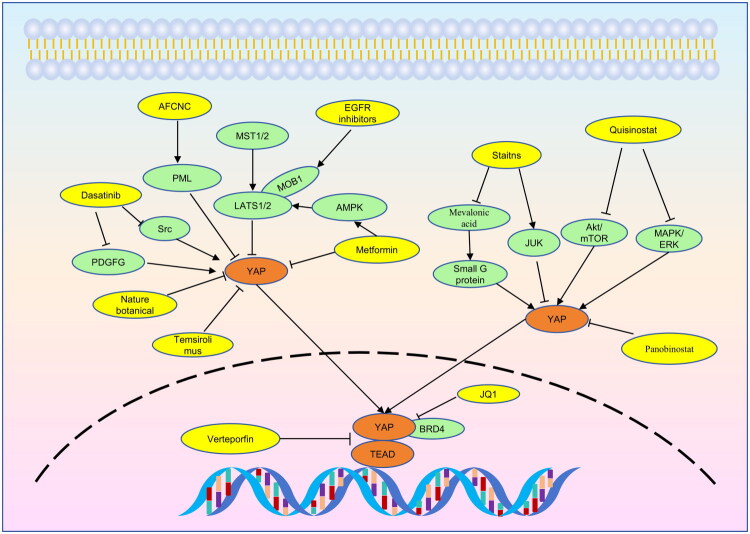
Targeting YAP for ESCC therapy. Dasatinib inhibits YAP activity through src and PDGFG. Nature botanical, Panobinostat, and temsirolimus can directly inhibit YAP activity. Metformnin can activate AMPK, resulting YAP inhibition by activating LATS1/2 and directly regulating YAP. AFCNC promotes YAP degradation by PML. Verteporfin disrupts the interaction between YAP and TEAD, leading to the transcription inhibition of YAP. Statins inhibit YAP through JUK and small G protein. EGFR inhibitor can block phosphorylation of MOB1, thus activating LATS1/2 and inactivating YAP. JQ-1 can inhibit transcription of YAP target genes. Quisinostat downregulates YAP activity through inhibiting akt/mTOR and MAPK/ERK signaling pathways.

The Hippo signaling pathway function as a mechanism that suppresses the growth of tumors and YAP is correlated with the occurrence and progression of ESCC. As a result, targeting Hippo/YAP provides potential opportunities for cancer therapy (Figure 6). Currently, many YAP-based inhibitors have been developed. Although they are not yet used clinically, they have shown good effects in ESCC cells and animal models.

### 6.1. Verteporfin

Disrupting the interaction between YAP and TEAD transcription factors would be an effective strategy as YAP can bind to TEAD and then activate target gene expression. Verteporfin is a photosensitizer that is used in photodynamic therapy. Many reports have demonstrated that verteporfin can block YAP-TEAD activity. Specifically, on the one hand, verteporfin can interfere with the interaction between YAP and TEAD, hence suppressing target gene expression including cyclin D1, cyclin E1, MMP2 [103,104]. On the other hand, verteporfin increases YAP cytoplasmic retention [105]. These findings imply that verteporfin, which targets YAP, could be a potential treatment drug for ESCC. However, the anti-tumor effects of verteporfin may extend beyond the inhibition of YAP-TEAD complexes, and it has been reported that the clinical effect of verteporfin in targeting YAP-TEAD is limited due to its off-target effects and cytotoxicity [106–108]. This has encouraged more efforts to develop allosteric inhibitors that disturb the YAP-TEAD connection. In addition, a study has shown that verteporfin inhibits ESCC cell survival and reverses resistance to paclitaxel *via* inducing ferroptosis, however, the study did not assess the impact of YAP on its effectiveness although emerging evidence has verified that YAP is closely related to ferroptosis [109]. Moreover, another study showed that verteporfin effectively prevents the progression and overcomes the paclitaxel chemoresistance of ESCC cells by preventing tumor angiogenesis [110].

### 6.2. Jq1

Zanconato et al. reported that YAP mediates transcriptional addiction in cancer cells through bromodomain-containing protein 4 (BRD4) which is a coactivator of the bromodomain and extraterminal domain (BET). JQ1, a BET inhibitor, inhibits BRD4 function and reduces the expression of genes controlled by YAP [111]. It has been reported that JQ1 can inhibit ESCC progression both *in vivo* and *in vitro* by targeting c-Myc, especially in c-Myc amplified or highly expressed xenografts [112]. Moreover, a recent study showed JQ1 could increase migration ability by promoting cell autophagy in KYSE-450 and KYSE-150 ESCC cell lines, which can be reversed by 3-methyladenine (autophagy inhibitor) [113]. In view of the above results, combining autophagy inhibitor and JQ1 may be a promising way for the ESCC treatment.

### 6.3. Arsenic compounds

Arsenic compounds have been shown to have a positive effect on the treatment of several cancers [114]. Zhou et al. developed arsenic-ferrosoferric oxide conjugated Nano Complex (As2S2–Fe3O4, AFCNC) and determined that AFCNC treatment not only reduces the invasion and migration capacity but also significantly abrogates the protective effects of YAP 5SA overexpression on cisplatin-induced apoptosis in KYSE-450 cells. Importantly, the combination of AFCNC with cisplatin treatment or radiation therapy effectively decreased the tumor size in the xenograft mouse model of ESCC. According to reports, it has been observed that PML and YAP have a physical interaction, and the degradation of PML caused by arsenic leads to the degradation of YAP in ESCC cells [115].

### 6.4. Natural botanical

In recent years, natural botanical anti-tumor drugs have received increasing interest from experts in China and other countries. For instance, Gallic acid (GA), a major component of T. chebula, has been reported to inhibit ESCC proliferation by targeting YAP both *in vivo* and *in vitro* [116]. In addition, a natural product called Shikonin, extracted from Radix Arnebiae, was discovered to exhibit anti-tumor effects by inhibiting YAP in gastric cancer and colon cancer cells [117,118]. Furthermore, *in vivo* and *in vitro* studies in ESCC showed Shikonin can inhibit tumor growth in ESCC, suggesting that Shikonin could be a potential drug for ESCC treatment [119].

### 6.5. Temsirolimus

Temsirolimus is a mTOR inhibitor approved by the US FDA in 2007 for treating advanced kidney cancer. Recently, a study reported that it can block YAP nuclear localization, thereby suppressing YAP activity in idiopathic pulmonary fibrosis, indicating that it may function as a YAP inhibitor [120]. One study demonstrated that temsirolimus treatment can reduce ESCC cell proliferation by inhibiting the activation of mTOR and its downstream effectors [121]. Furthermore, animal model assays showed temsirolimus successfully prolongs the survival of orthotopic esophageal cancer-bearing mice and markedly reduces the size of subcutaneous tumors in nude mice [121]. However, further clinical trials are needed to confirm its efficacy and safety in patients with ESCC.

### 6.6. Dasatinib

Dasatinib, a representative drug of Tyrosine kinase inhibitors, is widely used in chronic myeloid leukemia and mainly targets Bcr-Abl and Src [122]. YAP can be activated by Src *via* tyrosine phosphorylation [123,124]. Apart from inhibiting Src, dasatinib may also potently inhibit PDGFR, which is regarded as activating YAP [125]. There is limited studies on dasatinib in ESCC, with only reports showing that dasatinib enhances the sensitivity of ESCC cells to cisplatin [126]. However, it is worth noting that dasatinib has many side effects in clinical applications, such as chylothorax, pleural effusion, and pulmonary arterial hypertension. These side effects require close monitoring of relevant symptoms when dasatinib is administered.

### 6.7. Panobinostat and quisinostat

Panobinostat and quisinostat belong to Histone Deacetylase Inhibitors, and panobinostat was approved by the US FDA in 2015 for the management of relapsed/refractory multiple myeloma in combination with bortezomib and dexamethasone [127]. They can prevent the destruction of proteins by interfering with enzymatic activity of deacetylases. In addition, a clinical compound library screen demonstrated both panobinostat and quisinostat can reduce the expression of YAP in different cancer cell lines [128]. In ESCC, panobinostat inhibited ESCC cell proliferation by inducing cell cycle arrest, while a preclinical assessment showed that quisinostat’s anti-tumor effect is primarily due to blocking the Akt/mTOR and MAPK/ERK signaling pathways that are considered to activate YAP [129,130].

## 7. Clinical studies investigating YAP-targeting drugs in ESCC patients

In clinical practice, the greatest challenge for ESCC remains the treatment of patients with advanced or metastatic disease. Although several potential YAP inhibitors have been developed, their efficacy and safety in esophageal cancer still require validation through clinical trials. Here, we summarize some YAP-targeting drugs that have been verified in ESCC clinical studies (Table S1).

### 7.1. Metformin

Metformin is the primary medication used to treat type 2 diabetes clinically, and it has been shown to activate AMPK [131,132]. AMPK activation leads to the inhibition of YAP through the activation of LATS and the phosphorylation of YAP [54]. In addition, metformin was found to be related to the progression of ESCC by different studies. For example, Fan et al. reported that metformin reduces esophageal carcinogenesis in rats treated with NMBzA by blocking the AMPK/mTOR signaling pathway[133]. A similar study population-based cohort study showed metformin use decreases the risk of developing ESCC [134]. Furthermore, metformin can inhibit ESCC by downregulating PD-L1 expression *via* the IL-6 signaling pathway [135]. Overall, metformin may be a potential drug for the clinical treatment of ESCC.

### 7.2. Statins

Statins are used for both primary and secondary prevention of cardiovascular disease now[136,137]. Mechanistically, statins have been shown to inhibit HMG-CoA reductase, preventing the synthesis of mevalonic acid, a precursor of non-steroidal isoprenoids, which are lipid attachment molecules for small G proteins such as Ras, Rho, and Rac. Therefore, statins may limit isoprenoid production and prevent small G protein activation [138–140]. The activation of Rho is crucial for the nuclear accumulation of YAP through actin remodeling [38,141]. Furthermore, statins have been identified as inhibitors of YAP, effectively blocking YAP’s movement into the cell nucleus and its ability to initiate transcription [38,142–145]. Yuan et al. found that atorvastatin not only inhibits the cAMP and Rap1 signal pathways but also decreases phosphorylation levels of ERK^T185/Y187^, CDK1^T14^, and BRAC1^S1189^ *via* proteome and phosphoproteome analysis in ESCC cells [146]. Of note, YAP can be phosphorylated by the cell-cycle kinase CDK1 and ERK [147,148]. This indicates that YAP is at least partly involved in the functionality of statins in ESCC. Importantly, a clinical study conducted by Lin’s research team confirmed that the use of statins was associated with a reduced incidence of ESCC in patients who chew betel nuts [149]. Another cohort study that statin use (rosuvastatin, pravastatin, and simvastatin) was associated with better survival outcomes for patients with ESCC receiving concurrent chemoradiotherapy [150]. These findings suggest that statins could be an effective therapeutic choice for ESCC.

### 7.3. EGFR inhibitor

EGFR, one of the ERBB family tyrosine kinases, is a widely useful therapeutic target in different types of cancer, and EGFR inhibitors have been approved by the United States Food and Drug Administration for the treatment of non-small cell lung cancer and pancreatic cancer [151]. EGFR activation can cause the phosphorylation of MOB1, which inhibits LATS1/2 and activates YAP/TAZ [152]. In addition, EGFR is frequently amplified and overexpressed in ESCC and patients with EGFR overexpression exhibit a poor prognosis [153,154]. Furthermore, in clinical trial of ESCC, several studies have shown that EGFR inhibitors (erlotinib, icotinib, larotinib) display anti-tumor effects, especially in patients with EGFR overexpression [155–157].

## 8. Challenges and future directions in YAP-targeted therapy for ESCC

Despite significant progress in understanding the molecular mechanisms of ESCC, it remains the one of the most lethal cancers worldwide. YAP is closely related to the initiation and progression of ESCC and is becoming an attractive therapeutic target for ESCC. Nevertheless, certain obstacles remain unresolved. For example, the target genes of YAP and the mechanisms of activating YAP are yet totally understood.

Moreover, ESCC is known for its molecular and phenotypic heterogeneity, which poses a challenge in designing effective YAP-targeted therapies [158]. Future directions may involve identifying subtypes of ESCC with differential YAP dependency and tailoring therapies accordingly. Besides, although current reports on the use of spatial transcriptomics and CRISPR screening in YAP-targeted therapy for ESCC are limited, both technologies have shown promise in studying the tumor microenvironment and identifying therapeutic targets. In the future, CRISPR-Cas9 screening may help identify YAP co-oncogenes in ESCC. Furthermore, integrating spatial transcriptomics to analyze YAP’s spatial expression patterns in ESCC could support the development of precise targeting-YAP strategies. Interestingly, a recent single-cell sequencing study of ESCC samples has identified immune characteristics associated with neoadjuvant immunochemotherapy, revealing that LRRC15^+^ fibroblasts and SPP1^+^ macrophages are critical targets of treatment resistance [159]. Future combination therapies targeting these two factors, along with YAP, could offer new insights into ESCC treatment. In addition, many YAP inhibitors may have off-target effects or affect other signaling pathways, leading to unintended consequences [160]. Future research should focus on developing more specific YAP inhibitors with minimal off-target effects to enhance efficacy and reduce toxicity.

Additionally, due to its interaction with several signaling pathways, focusing just on targeting YAP may result in compensatory responses in the body, leading to the failure of treatment. Therefore, the simultaneous utilization of YAP inhibitors and other inhibitors targeting cancer signaling pathways is anticipated to enhance the treatment efficacy of ESCC.

Furthermore, YAP is intrinsically disordered with a high degree of structural flexibility [36,161]. There may be only a few regions within YAP that are susceptible to therapeutic inhibition, and the development of small molecule inhibitors is limited to those specific structures within the protein with a high degree of order [36]. Consequently, the drugs that are directly targeting YAP are still lacking to date. To address this challenge, Proteolysis Targeting Chimeras (PROTACs), a protein degradation technology, could provide new hope for solving this problem. Mechanically, PROTACs can specifically recruit E3 ubiquitin ligases and target proteins, thereby achieving the degradation of target proteins [162]. Of note, PROTACs has advantages over traditional small molecule inhibitors or antagonists, such as wider applicability, higher activity, better selectivity, higher safety, and lower drug resistance [163].

Finally, the utilization of natural chemicals that specifically target YAP could serve as a safe and efficient approach for treating cancer, owing to their inherent characteristics and minimal adverse reactions [164]. Going forward, new drugs targeting YAP are necessary and urgent to develop, and conducting large-scale clinical studies will speed up their translation into clinical practice. Overall, targeting YAP may provide new avenues for ESCC treatment.

## Supplementary Material

Supplementary Table 1.docx

## Data Availability

Data sharing is not applicable as no new data were created or analyzed in this study.
