# Supplementary material for: YAP as a therapeutic target in esophageal squamous cell carcinoma: insights and strategies
Source: Ann Med. 2025 Jul 22;57(1):2536200. doi: 10.1080/07853890.2025.2536200 (PMC12284994; doi:10.1080/07853890.2025.2536200)
Supplement: Supplementary Table 1.docx [file IANN_A_2536200_SM6263.docx]

Table S1. The clinical study of YAP-targeting drugs in ESCC.

| Inhibitor | Dosages | Efficacy | Toxicities | Reference |
| --- | --- | --- | --- | --- |
| Metformin | Low group (＜175 DDD), medium group (175–300 DDD), and high group (＞300 DDD) | The incidence rate of ESCC is 3.5 vs. 5.3 per 100,000 person-years in metformin users vs. nonusers, and metformin use significantly lowers ESCC incidence. | unknown | [134] |
| Statin | statin users group (≥28 cDDDs) and statin nonusers group (<28 cDDDs) | The incidence rate of ESCC is 2.03 vs. 3.02 per 100,000 person-years in statin users vs. nonusers, and statin use significantly reduces ESCC incidence. | unknown | [149] |
| Statin | statin users group (≥28 cDDDs) and statin nonusers group (0 cDDDs) | The adjusted HR for all-cause mortality is 0.65 (95% CI, 0.51–0.84; P = 0.0009) and for ESCC-specific mortality is 0.63 (95% CI, 0.47–0.84; P = 0.0016) in statin vs. non-statin users. Statin use confers the greatest survival benefits during chemoradiotherapy. | unknown | [150] |
| Larotinib | 350 mg per day | The overall response rate is 20%. The median overall survival and progression-free survival are 8.0 (95% CI 4.9–10.2) months and 3.4 (95% CI 2.4–3.7) months, respectively. Larotinib showes significant antitumor activity in clinical studies. | Diarrhea, rash, and palmar-plantar erythrodysesthesia syndrome, elevated AST/ALT, vomiting. | [156] |
| Erlotinib | 150 mg per day | The inclusion of erlotinib notably enhances overall survival of patients with ESCC (median, 39.4 versus 27.4 months; hazard ratio, 0.75; P = 0.025). | Rash | [157] |
| Icotinib | 250 mg, three times daily | In ESCC patients, the objective response rate is 16.7% (95% CI, 6.7%-26.6%), and the disease control rate is 46.3% (95% CI, 33.0%-59.6%). The median progression-free survival and overall survival are 52 (95% CI, 40-95) days and 153 (95% CI, 139-218) days, respectively. | Rash, diarrhea | [155] |
